# Effects of glycerol and sodium pentaborate containing new formulation on sleeve gastrectomy model in rats

**DOI:** 10.1590/ACB361105

**Published:** 2021-12-08

**Authors:** Mirkhalig Javadov, Emrah Karatay, Alev Cumbul, Suleyman Orman, Erhan Aysan

**Affiliations:** 1 MD. Assistant Professor. Yeditepe University Faculty of Medicine - Department of General Surgery - İstanbul, Turkey.; 2 MD. Marmara University Pendik Training and Research Hospital - Department of Radiology - İstanbul, Turkey.; 3 PhD. Yeditepe University Faculty of Medicine - Department of Histology and Embriology - İstanbul, Turkey.; 4 MD. Associate Professor. Yeditepe University Faculty of Medicine - Department of General Surgery - İstanbul, Turkey; 5 MD. Professor. Yeditepe University Faculty of Medicine - Department of General Surgery - İstanbul, Turkey.

**Keywords:** Gastrectomy, Glycerol, Rats

## Abstract

**Purpose:**

The development of cutting surface leakage and postoperative peritoneal adhesions (PPA) after sleeve gastrectomy (SG) are the most serious operative complications. We investigated the effectiveness of the newly developed glycerol and sodium pentaborate containing formulation on the prevention of these complications.

**Methods:**

Sixteen Sprague Dawley rats (mean weight 310 ± 50 g, mean age 3 months old) were divided into two groups, consisting of eight rats in each. SG and a double-layer suture technique were performed for each group. In study group, there was the mixture of 2 mL 3% glycerol plus 3% sodium pentaborate formulation, and in the control group 2 mL 0.9% NaCl was injected into the peritoneal cavity. Rats were sacrificed after 30 days, then macroscopic adhesion grade scoring and histopathological evaluations were assessed.

**Results:**

Macroscopic PPA scores in the control and study groups were 2.75 ± 0.16 and 1.50 ± 0.327, respectively (*p* = 0.004). Histopatologic fibrosis scores in the control and study groups were 0.87 ± 0.125 and 2.00 ± 0.26, respectively (*p* = 0.002).

**Conclusions:**

In SG operation model, glycerol plus sodium pentaborate compound decreased PPA formation and also increased stomach cut surface line fibrosis. This new formulation is hopeful for more safe SG operations.

## Introduction

Because of the popularity of obesity surgery, in the last five years the most common upper gastrointestinal surgical procedure has been sleeve gastrectomy (SG). Although there are different obesity surgery techniques, technical convenience and satisfying results have led to the fast implementation of SG. Low complication rates of SG are the other important cause of preference of this technique^1^.

Complications of SG include bleeding, leakage, metabolic problems, and postoperative peritoneal adhesions (PPA) related refractory vomiting. Operative complication rates are low, but when they occur morbidity and mortality rates are high according to metabolic deficiencies of morbid obese cases. The incidence of PPA related refractory vomiting is 1.5-5%, and it is the most common cause of prolonged hospitalization^2^. Refractory vomiting increases the risk of leakage, as it increases the pressure in the cut surface area.

Cut surface leakage after SG is the most feared complication with incidence of 0-7%^3^^-^ 8. Some different tools and techniques have been used to strengthen the cutting surface, such as glycolide-trimethylene carbonate copolymer, bovine pericardial strips, fibrin glue, amniotic membrane, protein-rich plasma, and various hemostatic agents^9^^-^ 12.

Glycerol is one of the most common molecules in living organisms. Fatty tissues consist of one molecule of glycerol combined with three molecules of fatty acids. Glycerol is used in medical, pharmaceutical, and personal care preparations, mainly as a means of improving smoothness, providing lubrication, and a hygroscopic substance^13^^,^ 14. We revealed the PPA reducing and colonic anastomosis healing effects of glycerol^15^^,^ 16.

Sodium pentaborate is the molecule derived from boron. Its wound healing and anti-inflammatory effects were demonstrated before^17^^-^ 19. We produced new formulation compounded with 3% glycerol and 3% sodium pentaborate and patented it. We demonstrated that this new formulation reduces PPA more effectively than glycerol by itself^20^.

In this study, we evaluated the effectiveness of the new formulation on the SG model in rats.

## Methods

This prospective study was approved by the Yeditepe University Animal Research Local Ethics Committee (IRB: 879/2020) and was performed at the Experimental Animal Production and Research Laboratory of Yeditepe University, Istanbul, Turkey. Study protocol was approved by the local Animal Ethics Committee. All protocols were in accordance with the regulations governing the care and use of laboratory animals in the Declaration of Helsinki.

Sixteen Sprague Dawley rats (mean weight 310 ± 50 g, mean age 3 months old) were used and randomly divided into two groups, consisting of eight rats in each. The animals were housed in stainless steel cages under controlled temperature (23°C) and humidity conditions, with 12-hour dark/light cycles. Rats were maintained on a standard laboratory diet with tap water ad libitum throughout the experiment, except for an overnight fast before surgery.

Before the operation, rats were fasting for 12 hours and, in order to avoid dehydration, 2-mL saline was delivered by subcutaneous injection. Surgical procedures were performed under aseptic conditions. Rats were anesthetized with 50 mg/kg ketamine hydrochloride (Alfamine^®^, Ege Vet, Izmir, Turkey) and 5 mg/kg xylazine (Rompun^®^, Biofarma Ilac Sanayi, Istanbul, Turkey) intraperitoneally. A 3-cm long midline abdominal incision was made to sufficiently expose the stomach. Greater curvature vessels were cut from the cardia to the pylorus, using the electrocautery.

Cutting line on the greatest curvature of the stomach was determined by using vascular forceps, and approximately 70% of the stomachs were resected in all rats. The cutting surfaces were stitched a double-layer suture technique with polyglactin 910 (Vicryl^®^ 5/0, Ethicon, Sao Paulo, SP, Brazil). In the control group, 2-mL sterile 0.9% NaCl was poured onto the cutting surface and spread into the peritoneal cavity. Same protocol was used in the study group with 2-mL sterile 3% glycerol plus 3% sodium pentaborate solution.

The laparotomies were closed with two-layer stitching technique: abdominal fasciae were sewn with continuous polyglactin 910 suture, and skins were sewn with continuous silk (Perma-Hand^®^ Silk suture 3/0, Ethicon, Sao Paulo, SP, Brazil) suture. Rats were kept in separated cages in three weeks and fed with standard pellet rat food. Except Sundays, animals were observed daily and the health status was evaluated. The rats were sacrificed by intraperitoneal 100-mg/kg sodium pentothal injection on postoperative day 30.

Long midline incisions were carried out for abdominal explorations. The PPAs were evaluated by an adhesion grading scoring system (Table 1). The stomachs were resected totally. Food residues in the lumen was cleaned by tap water. Cutting line was resected from other parts of the stomach and placed in 10% formalin for histopathological evaluation.

**Table 1:** Definitions of macroscopic adhesion grade scoring.

Grades	Definition
0	No adhesion
1	Self-separating adhesions
2	Adhesions separated by traction force
3	Adhesions separated by dissection

### Histopathologic evaluation

Tissue samples were immersed in 10% neutral formaldehyde in 0.1 M phosphate buffer (pH 7.4) for fixation at 40°C, followed by dehydration in an alcohol series, and embedded in paraffin. The paraffin-embedded sections of 5-μm thickness were prepared by sectioning the paraffin tissue blocks using a rotary microtome (Leica RM 2245 model; Leica Instruments, Germany). The tissue sections were mounted on poly-L-lysine coated slides. Then, tissues were stained with hematoxylin and eosin (H&E) and Masson’s trichrome (TCM) (Merck, Darmstadt, Germany). The experienced histologist who was blind to the groups graded the staining sections semi-quantitatively and evaluated them according to the histopathological fibrosis scoring system (Table 2).

**Table 2:** Definitions of histopathologic fibrosis grade scoring.

Grades	Definition
0	No fibrosis: no fibroblasts and/or collagen fibers
1	Mild fibrosis: few fibroblasts and/or collagen fibers
2	Moderate fibrosis: more fibroblasts and/or collagen fibers
3	Severe fibrosis: lots of fibroblasts and/or collagen fibers

### Statistical analysis

Statistical Package for the Social Sciences (SPSS) 22 was used for statistical analysis of the data. Results were evaluated with a confidence interval of 95% and p<0.05. Descriptive statistical methods (mean, standard deviation, minimum, and maximum) and independent t-test were used for control and study groups.

## Results

**Figure 1:**
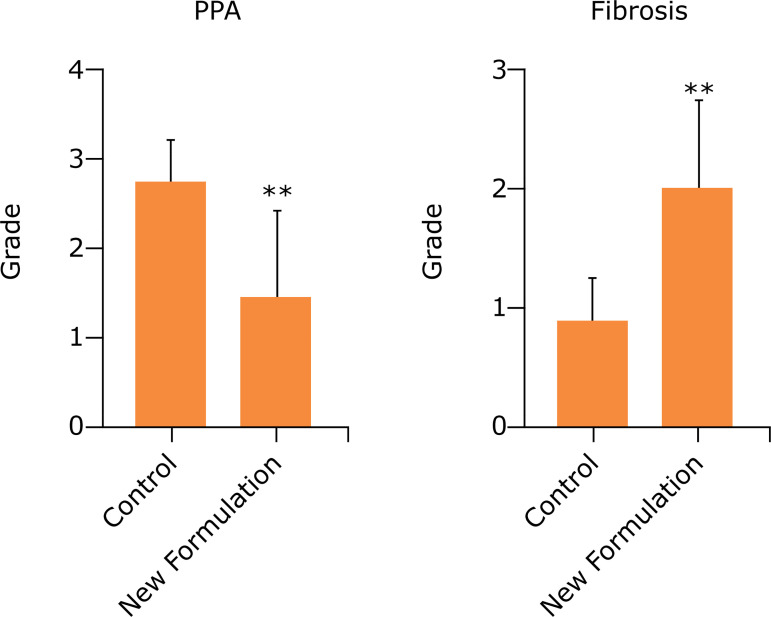
Comparison of macroscopic PPA and histopathologic fibrosis scores of the groups.

Macroscopic PPA scores in the control and study groups were 2.75 ± 0.16 and 1.50 ± 0.32, respectively (p = 0.004). Histopathologic fibrosis scores in the control and study groups were 0.87 ± 0.12 and 2.00 ± 0.26, respectively (p = 0.002) (Table 3, Fig. 1).

**Table 3:** Mean values of macroscopic PPA and histopathologic fibrosis scores of the groups.

	Control group (n=8)	New formulation group (n=8)	*p*
PPA score (mean ± SEM)	2.75 ± 0.16	1.50 ± 0.32	0.004
Fibrosis score (mean ± SEM)	0.87 ± 0.12	2.00 ± 0.26	0.002

PPA: postoperative peritoneal adhesion; SEM: scanning electron microscope.

PPA: postoperative peritoneal adhesion; **p <0.01.

**Figure 2:**
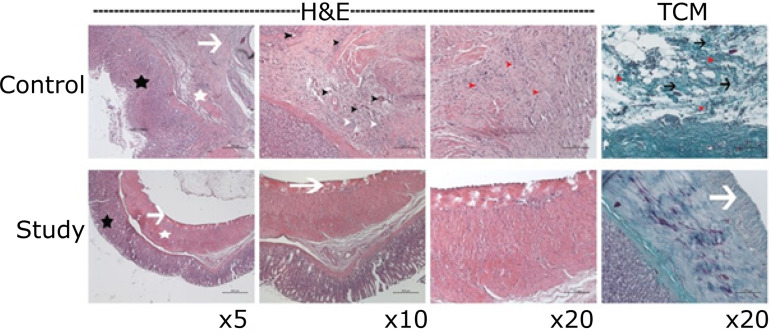
Comparison of microscopic histopathologic fibrosis scores of the control and study groups.

In Figs. 1 and 2, mucosal glands are indicated by black asterisk, muscle layer is indicated by white asterisk, tunica serosa is indicated by white arrow, new vessels are indicated by black arrowhead, infiltration in tunica submucosa is indicated by white arrowhead, new collagen fibers in tunica serosa are indicated by black arrow, and fibroblast activities is indicated by red arrowhead. In sections, new blood vessels, new collagen fibers, infiltration areas are increasing in study compared with control group. Sections have scale bars of 400, 200 and 100 μm. Images are x5, x10 and x20 magnifications.

H&E: hematoxylin-eosin; TCM: Masson’s trichrome.

## Discussion

Sleeve gastrectomy is the most performed procedure for surgical treatment of obesity^1^^,^ 25. Most often operative complications of SG are cut surface leakages, bleeding, and twisting^2^^-^ 4. Lots of twisting cases are related to PPA^5^^-^ 8.

Many techniques, methods, and materials such as amnion matrix patch, omental graft, fibrin glue, etc. were used to prevent these complications^9^^-^ 10. Unfortunately, despite of all attempts, these complications remain a common problem after obesity surgery^2^^-^ 6.

Our team has been focused on preventing PPA for many years and, for this purpose, evaluated various techniques, materials, and solutions^21^^-^ 24. We revealed that glycerol is highly effective in preventing PPAs^15^.

PPA reducing agents may be harmful for gastrointestinal anastomosis healing. Because of that, we investigated the effectiveness of glycerol on colonic anastomoses. In our study, we demonstrated that glycerol has a positive effect on colon anastomosis healing^16^.

The wound healing effects of various boron compounds have previously been demonstrated^17^^,^ 19. During in-vitro and in-vivo studies performed by our team, sodium pentaborate was shown to exert anti-inflammatory effects through cell proliferation, cell migration, and growth factor expression pathways and to accelerate wound healing in different wound models^17^^,^ 19 ^,^ 20. In a prospective randomized clinical trial, we also showed that sodium pentaborate gel can prevent radiation-induced dermatitis in breast cancer patients^18^.

Recently, we produced a new formulation: a compound of 3% glycerol and 3% sodium pentaborate. We evaluated the PPA-preventive effect of this formulation on the peritoneal adhesion model in rats and found a statistically significant decrease in PPAs. We concluded that our results are related to synergistic effect of sodium pentaborate and glycerol. Sodium pentaborate ensures anti-inflammatory and wound healing acceleration features, and glycerol ensures effective mechanical separation of the wound healing environment^20^.

In this study, we evaluated PPA by an adhesion grading scoring system (Table 1). SG cut surface healing was evaluated by the histopathologic fibrosis scoring of the anastomotic line (Table 2). We found that the new formulation created a statistically and significantly lower amount of PPA compared to the control group (p=0.004). The histopathologic fibrosis score of the new formulation group was statistically and significantly higher than the control (p=0.002) (Table 3, Fig. 1).

This situation can be explained by the difference in the main functions of glycerol and sodium pentaborate. While glycerol separates the peritoneal surfaces, sodium pentaborate accelerates wound healing activity via fibroblast collagen transformation and deposition of collagen at the anastomosis line. Since PPA is a form of collagen deposition, it may be thought that this property of sodium pentaborate would increase the amount of PPA, whereas the amount of PPA in fact decreased in the group using the new formulation. We hypothesized that this incongruity might be explained by the synergistic effects of glycerol and sodium pentaborate.

Again, all these results exhibit the positive effects of glycerol and sodium pentoborate mixture on cut surface healing with PPA inhibition.

In this study, we revealed that glycerol and sodium pentaborate compound either decreases PPA formation or by the way of increasing stomach cuts surface line fibrosis.

## Conclusion

These results are promising for the safety of SG surgeries, as they reduce the two major complications of PPA and cut surface leakage. It is planned to start phase I clinical study after obtaining clinical use permissions and human ethics committee approval for our formulation.
